# The treatment of periarticular soft tissue sarcoma following neo-adjuvant radiotherapy: a cohort study

**DOI:** 10.1186/s12957-015-0515-8

**Published:** 2015-03-14

**Authors:** Carl M Green, Nam Nguyen, James Wylie, Ananya Choudhury, Jonathan J Gregory

**Affiliations:** Manchester Royal Infirmary, Oxford Road, Manchester, M13 9WL UK; The Christie Hospital, 550 Wilmslow Road, Manchester, M20 4BX UK

**Keywords:** soft tissue sarcoma, radiotherapy, surgery, outcome, pre-operative, neo-adjuvant, articular, joint, jcomplications

## Abstract

**Background:**

Optimising post-operative joint function is challenging when treating periarticular soft tissue sarcoma (STS). Radiotherapy reduces local recurrence rates but periarticular fibrosis may adversely affect joint function. Neo-adjuvant radiotherapy requires lower doses and smaller treatment volumes and therefore has potential benefits for the management of periarticular STS, but has previously been shown to be associated with an increased risk of post-operative wound complications. This study assesses initial outcome and complications after treatment with neo-adjuvant radiotherapy and surgery for patients with periarticular STS.

**Methods:**

Seventeen patients (mean age 52.5 years) were treated using a standard protocol between January 2009 and June 2012 with three-dimensional conformal neo-adjuvant radiotherapy to a dose of 50 Gy in 25 fractions at a single centre, followed by limb salvage surgery. Patients were assessed weekly for adverse effects during radiotherapy. Surgery was planned for 6 weeks following completion of radiotherapy. Patients remain under follow-up with regular Toronto Extremity Salvage Scores (TESS) performed.

**Results:**

No patients had a significant adverse effect during radiotherapy. Three patients (17.6%) suffered a wound complication following surgery, all treated conservatively. Magnetic resonance imaging (MRI) demonstrated a reduction in mean maximal tumour diameter from 7.56 to 5.24 cm (*p* = 0.017, 11 of 17 patients). Tumour necrosis was measured between 50% and 100% in 10 of 11 resections where accurate assessment was possible. One patient had further surgery due to incomplete margins. No patients required post-operative radiotherapy. No local recurrences have occurred after a mean follow-up of 32 months (range 19 to 59 months). Two patients have developed metastatic disease. Mean TESS scores for upper and lower limb patients were 98.5 and 85.5, respectively, at latest follow-up.

**Conclusions:**

We have demonstrated improved wound complication rates compared to the existing literature on the use of neo-adjuvant radiotherapy. This may relate to modification of the technique and patient selection compared to previous series. Excellent functional outcomes can be obtained with this treatment strategy.

## Background

Soft tissue sarcoma (STS) includes a heterogenous group of rare tumours which account for approximately 1% of all adult malignancies in the UK, equivalent to an incidence of 37 cases per million per year [1]. STS are tumours of mesodermal origin and are classified according to their tissue of origin, the most common types being myxofibrosarcoma, liposarcoma, and leiomyosarcoma [2]. STS are prone to local recurrence despite optimal treatment, and intermediate or high-grade tumours have significant metastatic potential [3].

Surgery is the primary treatment modality for STS with the intention to remove the tumour fully whilst preserving function. Periarticular tumours, defined as being related to the metaphysis or epiphysis of a long bone, pose a particular problem to the surgeon due to the proximity of an articulation and neurovascular structures. Maximising functional outcome at these sites is challenging.

Radiotherapy reduces local recurrence rates and is indicated in the treatment of tumours which are high grade, deep to fascia and >5 cm in largest diameter [4,5]. The use of adjuvant versus neo-adjuvant radiotherapy is controversial. Adjuvant, or post-operative, radiotherapy has been demonstrated to achieve similar local control rates and survival to radical resection [4] by using a recommended dose of 60 to 66 Gy in 2 Gy fractions [5]. The radiation field covers the estimated tumour bed, a 25 mm circumference for clearance, the surgical incision, and drain sites. Treating patients with adjuvant radiotherapy as opposed to neo-adjuvant radiotherapy has been linked to a higher risk of developing acute toxic skin effects (68% *vs*. 36% of patients), as well as being linked to an increased likelihood of developing joint stiffness, subcutaneous fibrosis and soft tissue oedema [6,7].

Neo-adjuvant, or pre-operative, radiotherapy is less commonly used to treat STS in the UK. The standard regimen for neo-adjuvant radiotherapy is 50 Gy in 2 Gy fractions with surgery 6 weeks after completion, hence reducing the dose required for each patient [8]. Patients treated with neo-adjuvant radiotherapy have been documented to have a better long-term functional outcome, a higher likelihood for negative margins on resection and a lower risk of developing joint stiffness [6,7,9]. However, concerns with regard to wound complications remain, as studies by O’Sullivan *et al.* and Cannon *et al*. have demonstrated a wound complication rate of 34% to 35% of those treated with neo-adjuvant radiotherapy as opposed to 16% to 17% for adjuvant radiotherapy [6,10]. Currently, neo-adjuvant radiotherapy in STS management is most commonly used if the size of radiation field for post-operative treatment is likely to be associated with significant late morbidity or if the tumour is of borderline operability [7,9,11]. In addition, neo-adjuvant radiotherapy is advantageous for certain radiosensitive histological subtypes (for example, myxoid liposarcoma) given the degree of tumour necrosis that can be achieved [12].

Few studies in the literature assess management strategies for periarticular tumours, and none assess the use of neo-adjuvant radiotherapy in the treatment of periarticular STS. Turcotte *et al.* (2009), Rudiger *et al.* (2007) and Pritsch *et al.* (2007) describe groups of patients treated for soft tissue sarcoma involving the popliteal fossa [13-15]. However, patients in these studies were treated with varying management plans, including primary amputation in 14% to 28% of patients, with some patients being treated for secondary disease. Although wound complication rates (17% to 27%) and local recurrence (5.6% to 10.3%) are documented, it is difficult to interpret these results in relation to primary disease only.

This study aims to assess the initial results, in particular wound complications, of patients with periarticular soft tissue sarcoma treated with neo-adjuvant radiotherapy. Secondly, we aim to specifically assess the functional outcome of these patients as it would be expected that their functional outcome for tumours at these sites would differ from ‘standard’ diaphyseal sarcomas.

## Methods

**Figure 1:**
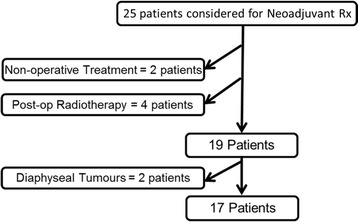
**Patients were identified using a database which provides details of those discussed for suitability for neo-adjuvant radiotherapy.**

A total of 25 patients were identified using a database which provides details of those discussed for suitability for neo-adjuvant radiotherapy for a diagnosed soft tissue sarcoma of the extremity between January 2009 and June 2012 (Figure 1). A periarticular STS was defined as a sarcoma in which part of the tumour was superficial to the joint, that is overlying the epiphysis or metaphysis of the bone. There were some large tumours in the study that covered part of the diaphysis in addition to the metaphysis. Tumours which were only overlying the diaphysis were excluded.

The rationale for the use of neo-adjuvant radiotherapy is to reduce the radiotherapy dose and field with the intention of optimising post-operative function. Neo-adjuvant radiotherapy was not thought to be appropriate in patients with very rapidly growing tumours (which may become inoperable if growth continued through radiotherapy) if limb salvage was not felt to be possible or if significant pre-existing skin problems rendered the risk of post-operative wound complications unacceptable. Tumours in the groin and the proximal adductor compartment were not considered due to the high risk of wound complications seen with tumours at this site. Tumours in the distal adductor compartment were considered for pre-operative radiotherapy. In cases felt not to be suitable for neo-adjuvant radiotherapy, conventional management was employed utilising surgery and adjuvant radiotherapy.

Retrospective case note analysis was then conducted by a single clinician. Details of the patient referral, investigations, diagnosis and management were collected and analysed.

All patients were treated with neo-adjuvant three-dimensional conformal radiotherapy, delivered at a single centre with a dose of 50 Gy in 25 fractions of 2 Gy over 5 weeks treating the radiologically identified tumour and peri-tumour oedema seen on pre-operative magnetic resonance imaging (MRI) with a margin of 2 to 3 cm. Patients were assessed weekly by a clinician for adverse effects. Surgery was scheduled at 6 to 8 weeks after completion of radiotherapy. Patients were then seen at regular intervals by both surgeons and oncologists for follow-up. Wounds were assessed at 2 weeks, 6 weeks and 3 months post-operatively by the surgeon, with subsequent checks at 6 and 12 months before moving to annual follow-up. Cast immobilisation was required for two patients due to removal of the bone involved in the tumour for a total of 6 weeks, and a cricket pad splint was required for 4 weeks for a patient who required hamstring reconstruction.

The primary outcome measure was the development of a wound complication, with secondary outcome measures of functional outcome, local control and metastasis also assessed. Wound complications were regarded as minor (conservative management only of ≤6 weeks duration) and major (requiring operative debridement or soft tissue reconstruction, or prolonged conservative management). Statistical testing for differences in tumour size was made using a two-tailed student *t*-test with a *P*-value of <0.05 considered to be indicative of significance.

## Results

Seventeen patients (10 males, 7 females) with a mean age of 52.5 years were treated for periarticular STS between January 2009 and June 2012. All patients were able to complete the course of neo-adjuvant radiotherapy without any significant adverse events. One patient had a delay in surgical excision by 6 weeks due to excessive local erythema. Fifteen patients underwent primary wound closure at the time of index surgery, with two patients requiring a split skin graft (SSG) 7 days after initial surgery to complete wound closure. Three patients (17.6%) suffered from a wound complication post-operatively, all of which were treated conservatively (Table 1). There was no correlation between tumour size and wound complication.

**Table 1:** **Three patients (17.6%) suffered from a wound complication post-operatively, all of which were treated conservatively**

**Gender**	**Age**	**Diagnosis**	**Site of tumour**	**Complication**	**Management**	**Duration**
M	83	Myxofibrosarcoma	Right forearm/wrist	Dehiscence part of SSG	Secondary intention, antibiotics	5 weeks
M	54	Myxoid Liposarcoma	Left calf/ankle	Wound dehiscence	Secondary intention, antibiotics	4 months
F	77	Spindle Cell Sarcoma	Left wrist/forearm	Superficial infection	Oral augmentin	1 week

The initial mean maximal tumour diameter was 8.0 cm on MRI scan prior to radiotherapy. Eleven of 17 patients had a further MRI scan between completion of radiotherapy and surgical treatment, demonstrating a significant reduction in mean greatest dimension of tumour size from 7.56 to 5.24 cm (*P* = 0.017). This correlated well with a mean maximal tumour diameter of 5.75 cm on histology. Necrosis of 50% to 100% was demonstrated in 10 of 11 tumours in which it was possible to make an accurate assessment (Table 2). Seven tumours showed tumour necrosis of over 90%.

**Table 2:** **Necrosis of 50% to 100% was demonstrated in 10 of 11 tumours in which it was possible to make an accurate assessment**

**Gender**	**Age**	**Diagnosis**	**Site of tumour**	**Staging**	**Minimum margin**	**Margin**	**Necrosis**
M	48	Myxoid liposarcoma	Right knee	T2bN0M0	1 mm	Negative	100%
F	41	Myxoid liposarcoma	Left leg ant/lat	T2bN0M0	0.5 mm	Negative	
M	83	Myxofibrosarcoma	Right forearm/wrist	T1aN0M0	2 mm	Negative	98%
F	57	Malignant fibrous tumour	Right popliteal fossa	T2bN0M0	1 mm	Negative	
F	42	Myxoid liposarcoma	Right popliteal fossa	T2bN0M0	>5 mm	Negative	95%
M	57	Extraskeletal myxoid chondrosarcoma	Left wrist/forearm	T1bN0M0	0.5 mm	Negative	
M	54	Myxoid liposarcoma	Left calf/ankle	T2bN0M0	2 mm	Negative	100%
M	31	Sclerosing epithelioid fibrosarcoma	Left medial knee/thigh	T2bN0M0	1 mm	Negative	
M	59	Extraskeletal myxoid chondrosarcoma	Left knee	T2bN0M0	0.5 mm	Negative	
M	50	Synovial sarcoma	Left thigh/knee	T2bN0M0	0 mm	Positive	75%
M	31	Pleomorphic liposarcoma	Left anterior thigh/knee	T2bN0M0	3 mm	Negative	0%
F	77	Spindle cell sarcoma	Left wrist/forearm	T2bN0M0	3 mm	Negative	99%
F	37	Leiomyosarcoma	Right triceps/elbow	T2bN0M0	0.5 mm	Negative	50%
M	46	Myxoid liposarcoma	Left posterior knee/thigh	T2bN0M0	1 mm	Negative	100%
M	54	Pleomorphic myxofibrosarcoma	Right elbow	T2bN0M0	>5 mm	Negative	100%
F	75	Pleomorphic sarcoma	Left lateral shoulder	T2bN0M0	>3 mm	Negative	50%
F	51	Myxoid liposarcoma	Right posteromedial thigh	T2bN0M0	1 mm	Negative	

Negative surgical margins were achieved in all but one patient who histologically was found to have a single breach of the tumour capsule (Table 1); following multi-disciplinary team (MDT) discussion, a repeat resection was performed and no evidence of residual tumour was demonstrated histologically. No patients received any additional post-operative radiotherapy.

Toronto Extremity Salvage Scores (TESS) were obtained for 16 of 17 patients following treatment (Table 3). TESS improved over time in all cases, either due to an expected increase in score as follow-up progressed, due to recovery of a radial nerve neuropraxia with one patient (from 47 to 97.2), or after reconstructive surgery following tumour resection for two patients.

**Table 3:** **Toronto Extremity Salvage Scores (TESS) were obtained for 16 of 17 patients following treatment**

	**Mean TESS**			
	**1st score**	**Time from op**	**2nd score**	**Time from op**
Upper limb	86.1 (47 to 100)	13.4 months (7 to 29)	98.5 (97.5 to 100)	23.4 months (17 to 39)
Lower limb	77.2 (39.5 to 99.2)	18.3 months (3 to 44)	85.5 (53.1 to 100)	28.3 months (13 to 54)
Total	80.9	16.7 months	89.1	26.7 months

Sixteen patients remain under active follow-up, as one patient died with myxofibrosarcoma of the forearm died after an unrelated small bowel perforation. After a median follow-up of 27 months (range 19 to 59 months), no local recurrences have been diagnosed. One patient developed metastatic lymphadenopathy of the axilla treated with resection (now disease free), and one patient has developed pulmonary metastatic disease being treated palliatively.

## Discussion

Our study shows that the use of three-dimensional conformal radiotherapy given between 6 and 8 weeks prior to surgery is associated with a wound complication rate of 17.6% which is comparable to the level of wound complication documented for post-operative, or adjuvant, radiotherapy. Patients in our study were treated for tumours in anatomically challenging areas which would be assumed to have a high rate of complications. We have demonstrated that excellent functional outcome can be obtained for periarticular soft tissue sarcomas treated with neo-adjuvant radiotherapy and subsequent limb salvage surgery.

The use of neo-adjuvant versus adjuvant radiotherapy is a controversial issue in the management of STS [3]. Although increased wound complications with the use of neo-adjuvant radiotherapy are documented in the literature, benefits include better long-term function and potentially patient survival rates [4,6,7,9].

Our study shows that three patients (17.6%) developed a wound complication and none required operative management. One patient was treated with a week-long course of oral antibiotics to treat a minor infection after resection of a tumour overlying the wrist. One patient developed a minor infection and dehiscence of a small area of a split skin graft following resection of a myxofibrosarcoma overlying the right wrist and was treated with a 1-week course of antibiotics and 5 weeks of supervised dressings. One wound complication was classified as major due to protracted conservative management, which took 4 months to heal: this patient had excision of a myxoid liposarcoma overlying the left ankle. The surgical wound appeared to be healing initially but then broke down between day 14 to 21 post-surgery and dehisced. The wound required treatment with a negative pressure dressing (VAC) and a two of courses of oral antibiotics. No inpatient or operative treatment was undertaken.

O’Sullivan *et al*. and Cannon *et al*. show a higher wound complication rate in patients treated with neo-adjuvant radiotherapy [6,10]. O’Sullivan *et al*.’s land mark paper (2002) randomised patients to neo-adjuvant or adjuvant radiotherapy with the primary end point of a wound complication (infection or repeat procedure) within 120 days of surgery. Of 88 patients, 31 (35%) in the neo-adjuvant group compared to 16 of 94 patients (17%) in the adjuvant group suffered a wound complication, with lower limb tumours of both groups being most common. Wound complication rates from a study by Cannon *et al*. were similar (34% neo-adjuvant *vs*. 16% adjuvant) [10]. There was a higher proportion of patients with tumours of the thigh in these studies compared to our own, in which a higher complication rate was noted when compared to other anatomical sites (45% neo-adjuvant and 28% adjuvant) [6]. However, in O’Sullivan *et al*.’s study, patients received surgery between 3 and 6 weeks following completion of neo-adjuvant radiotherapy, with a 5-cm margin proximal and distal to the tumour included in the radiation field [6]. We scheduled patients for surgery 6 to 8 weeks after completion of three-dimensional conformal radiotherapy and margin of 2 to 3 cm, with none of our patients receiving a post-operative boost of radiotherapy. Baldini *et al*. demonstrated that flap closure or SSG is associated with increased risk of wound complication; primary closure of the wound was achieved for 15 of 17 patients (88.2%) in our study, compared to 58 of 88 (66%) in O’Sullivan *et al*.’s paper and 206 of 269 patients (77%) in Cannon *et al*.’s study [6,10,16]. Finally, we delivered radiotherapy in the modality of three-dimensional conformal radiotherapy, which was not performed in these previous studies. These factors may explain the improved wound complication rate in our series.

This study describes a set protocol of management for periarticular STS, based on neo-adjuvant radiotherapy and limb salvage surgery, performed in a single centre. There are very few articles in the literature describing the treatment of patients with a soft tissue sarcoma close to an articulation. Turcotte *et al*. Rudiger *et al*. and Pritsch *et al*. all described small groups of patients treated for STS of the popliteal fossa with most receiving limb salvage surgery [13-15], but treatment was varied and it is therefore difficult to assess the results of these papers in detail. Wound complication rates therefore vary; Pritsch *et al.* quotes a total wound complication rate of 23.1% (6 of 26 patients) with two patients requiring surgical debridement, and Rudiger *et al*. quotes a radiotherapy-related wound complication rate of 26.7% (4 of 15 patients). However, with both studies, it is unclear which patients received neo-adjuvant or adjuvant radiotherapy. Our paper therefore improves the literature with regard to the management of periarticular STS.

Other parameters of success of treatment include clearance of tumour, local control and long-term function. A reduction of mean maximal diameter of tumour in this group of patients was demonstrated, which has been noted in previous studies involving STS and in particular myxoid liposarcoma [17,18]. Following neo-adjuvant radiotherapy, 16 of 17 patients (94.1%) had clear margins on histological analysis. Zagars *et al*. also demonstrated that patients treated with neo-adjuvant radiotherapy showed a lower rate of positive surgical margins (86% *vs*. 67%) and post-radiotherapy complications [9].

With regard to function, three patients initially recorded low TESS; one patient was only 3 months after surgery, one patient suffered a transient wrist drop and one patient required excision of their quadriceps tendon. Two of these patients have made a significant improvement during their post-operative recovery, with a mild improvement noted for the third patient. However, with mean TESS scores for upper limb and lower limb tumours being 98.5 (mean 23.4 months) and 85.5 (mean 28.3 months), respectively, excellent functional outcomes have been obtained.

Although these results presented are promising, this study is not without limitation. The sample size is small, and hence, it is difficult to assess whether age, gender, tumour site and grade affect patient function, local recurrence and survival. Eleven of our 17 patients had an MRI scan between completion of radiotherapy and prior to surgery; this was highlighted within the department on completion of this study, and it is now standard practice in our unit to repeat the MRI scan after completion of radiotherapy. A limitation of the study is the absence of a control group of patients with periarticular tumours managed with surgery and adjuvant radiotherapy. The aim of this study was not to compare radiotherapy in adjuvant and neo-adjuvant settings as large studies have done this. The intention was to demonstrate that the wound complication rate with this technique is lower than previous studies of neo-adjuvant radiotherapy have reported. An acceptable wound complication rate allows this technique, with the associated benefits of reduced radiotherapy dosage, to be more widely adopted. Although the primary end point for this study was the rate of wound complication, follow-up continues and this will identify the local control rate.

Further studies are required with regard to delivery of radiotherapy. The VORTEX trial, a prospective randomised trial comparing the use of large, extended radiotherapy volumes with a smaller radiotherapy volume for adjuvant radiotherapy [5] has recently closed. The aim of this national UK trial is to assess if smaller volumes of radiotherapy affect functional outcome without compromising on local recurrence or patient survival. Results from this study may inform a future trial involving the use of neo-adjuvant radiotherapy.

## Conclusions

In conclusion, the primary outcome measure, wound complication rate, was better than the rate previously identified in the literature. If equivalent wound complication rate, local control and patient survival can be achieved with a lower radiation dose compared to adjuvant radiotherapy, this should confer a benefit to the patient.

## References

[1] Jemal A, Siegel R, Ward E, Murray T, Xu J, Thun MJ (2007). Cancer statistics 2007. CA Cancer J Clin.

[2] Shmookler B, Bickels J, Jelinck J, Sugarbaker P, Malawer M (2001). Bone and Soft-Tissue Sarcomas: Epidemiology, Radiology, Pathology and Fundamentals of Surgical Treatment. Musculoskeletal Cancer Surgery. Treatment of Sarcomas and Allied Diseases.

[3] Coindre JM, Terrier P, Guillou L, Le Doussal V, Collin F, Ranchere D (2001). Predictive value of grade for metastasis development in the main histologic types of adult soft tissue sarcomas: a study of 1240 patients from the French Federation of Cancer Centers sarcoma group. Cancer.

[4] Yang JC, Chang AE, Baker AR, Sindelar WF, Danforth DN, Topalian SL (1998). Randomized prospective study of the benefit of adjuvant radiation therapy in the treatment of soft tissue sarcomas of the extremity. J Clin Oncol.

[5] VORTEX Trial Management Group. Randomised trial of volume of post-operative radiotherapy given to adult patients with extremity soft tissue sarcoma. Version 6, 2010. [http://www.birmingham.ac.uk/research/activity/mds/trials/crctu/trials/vortex/index.aspx]

[6] O'Sullivan B, Davis AM, Turcotte R, Bell R, Catton C, Chabot P (2002). Preoperative versus postoperative radiotherapy in soft-tissue sarcoma of the limbs: a randomised trial. Lancet.

[7] Davis AM, O’Sullivan B, Turcotte R, Bell R, Catton C, Chabot P (2005). Late radiation morbidity following preoperative versus postoperative radiotherapy in extremity soft tissue sarcoma. Radiother Oncol.

[8] Grimer R, Judson I, Peake D, Seddon B (2010). Guidelines for the management of soft tissue sarcomas. Sarcoma.

[9] Zagars GK, Ballo MT, Pisters PWT, Pollock RE, Patel SR, Benjamin RS (2003). Preoperative vs. postoperative radiation therapy for soft tissue sarcoma: a retrospective comparative evaluation of disease outcome. Int J Radiat Oncol Biol Phys.

[10] Cannon CP, Ballo MT, Zagars GK, Mirza AN, Lin PP, Lewis VO (2006). Complications of combined modality treatment of primary lower extremity soft-tissue sarcomas. Cancer.

[11] Davis AM, O'Sullivan B, Bell RS, Turcotte R, Catton CN, Wunder JS (2002). Function and health status outcomes in a randomized trial comparing preoperative and postoperative radiotherapy in extremity soft tissue sarcoma. J Clin Oncol.

[12] Canter RJ, Martinez SR, Tamurian RM, Wilton M, Li CS, Ryu J (2010). Radiographic and histologic response to neo-adjuvant radiotherapy in patients with soft tissue sarcoma. Ann Surg Oncol.

[13] Turcotte RE, Ferrone M, Isler MH, Wong C (2009). Outcomes in patients with popliteal sarcomas. Can J Surg.

[14] Rüdiger HA, Beltrami G, Campanacci DA, Mela MM, Franchi A, Capanna R (2007). Soft tissue sarcomas of the popliteal fossa: outcome and risk factors. Eur J Surg Oncol.

[15] Pritsch T, Bickels J, Winberg T, Malawer MM (2007). Popliteal sarcomas: presentation, prognosis, and limb salvage. Clin Orthop Relat Res.

[16] Baldini EH, Lapidus MR, Wang Q, Manola J, Orgill DP, Pomahac B (2013). Predictors for major wound complications following preoperative radiotherapy and surgery for soft-tissue sarcoma of the extremities and trunk: importance of tumor proximity to skin surface. Ann Surg Oncol.

[17] Chung PWM, Deheshi BM, Ferguson PC, Wunder JS, Griffin AM, Catton CN (2009). Radiosensitivity translates into excellent local control in extremity myxoid liposarcoma. A comparison with other soft tissue sarcomas. Cancer.

[18] Roberge D, Skamene T, Nahal A, Turcotte RE, Powell T, Freeman C (2010). Radiological and pathological response following pre-operative radiotherapy for soft-tissue sarcoma. Radiother Oncol.

